# Good science is good science: we need specialists, not sects

**DOI:** 10.1007/s10654-020-00651-2

**Published:** 2020-06-20

**Authors:** Marc Lipsitch

**Affiliations:** Center for Communicable Disease Dynamics, Department of Epidemiology, Harvard T.H. Chan School of Public Health, Boston, MA USA

The Brazilian–British biologist Peter Medawar won the Nobel Prize in 1960 for his study of acquired immune tolerance. Beyond his scientific work, he was also a gifted writer and expositor of scientific culture. One of the many treasures of his *Advice to a Young Scientist* (1979) is a passage in his chapter on “Aspects of Scientific Life and Manners” where he discusses the techniques used in the hope of enlarging one’s reputation as a scientist or diminishing the reputation of others by nonscientific means.”

One such “trick,” Medawar writes, “is to affect the possession of a mind so finely critical that no evidence is ever quite good enough (‘I am not very happy about….’; ‘I must say I am not at all convinced by….’).” After all, as he writes in a different passage, “no hypothesis in science and no scientific theory ever achieves… a degree of certainty beyond the reach of criticism or the possibility of modification” [1].

I share Medawar’s pragmatic vision of scientific reasoning. Scientists must resist the temptation to excessive skepticism: the kind that says no evidence is ever quite good enough. Instead they should keep their eyes open for any kind of information that can help them solve problems. Deciding, on principle, to reject some kinds of information outright, or to consider only particular kinds of studies, is counterproductive. Instead of succumbing to what Medawar calls “habitual disbelief,” the scientist should pursue all possible inputs that can sharpen one’s understanding, test one’s preconceptions, suggest novel hypotheses, and identify previously unrecognized inconsistencies and limitations in one’s view of a problem.

This conception of science leads me to disagree with some elements of the philosopher of medicine Jonathan Fuller’s recent essay [2] about two sects within epidemiology, defined by what kinds of evidence they consider meaningful and how they think decisions should be made when evidence is uncertain. Fuller sees in the contrast two “competing philosophies” of scientific practice. One, he says, is characteristic of public health epidemiologists like me, who are “methodologically liberal and pragmatic” and use models and diverse sources of data. The other, he explains, is characteristic of clinical epidemiologists like Stanford’s John Ioannidis, who draw on a tradition of skepticism about medical interventions in the literature of what has been known since the 1980s as “evidence-based medicine,” privilege “gold standard” evidence from randomized controlled trials (as opposed to mere “data”), and counsel inaction until a certain ideal form of evidence—Evidence with a capital E—justifies intervening.

Fuller rightly points out that this distinction is only a rough approximation; indeed, there are many clinical epidemiologists who do not share the hardline skepticism associated with the most extreme wing of the evidence-based medicine community. But the distinction is also misleading in a subtle way. If the COVID-19 crisis has revealed two “competing” ways of thinking in distinct scientific traditions, it is not between two philosophies of *science* or two philosophies of *evidence* so much as between two philosophies of *action*. In March, as health systems in Wuhan, Iran, and Northern Italy teetered under the weight of COVID-19 cases, Ioannidis cautioned that we really didn’t know enough to say whether a response was appropriate, warning of a “once-in a-century evidence fiasco” and suggesting that the epidemic might dissipate “on its own” [3].

(I replied to that argument, explaining why we do know enough to act decisively against this pandemic [4]). To my knowledge, Ioannidis has never stated that early interventions should have been avoided, but by repeatedly criticizing the evidence on which they were based, he gives that impression.

On the question of how we interpret evidence, Fuller concludes that to understand the scientific disagreements being aired about COVID-19, we need to blend the insights of each camp. “Cooperation in society should be matched by cooperation across disciplinary divides,” he writes. I don’t understand what this kind of bothsidesism means when one side is characterized as accepting many types of evidence, and the other as insisting on only certain kinds. On the question of how we should make decisions under uncertainty, of course more data are better. But decisions are urgent and must be made with the evidence we’ve got.

This is not to deny that there are different and valuable perspectives on epidemiology. Like any other field, there are many specialties and subspecialties. They have different methods for how they study the world, how they analyze data, and how they filter new information. No one person can keep up with the flood of scientific information in even one field, and specialization is necessary for progress: different scientists need to use different approaches given their skills, interests, and resources. But specialization should not lead to sects—in this case, a group of scientists who accept only certain kinds of evidence and too rigidly adhere to a philosophy of non-interventionism.

Infectious disease epidemiologists must embrace diverse forms of evidence by the very nature of their subject. We study a wide range of questions: how and under what conditions infectious diseases are transmitted, how pathogens change genetically as they spread among populations and across regions, how those changes affect our health, and how our immune systems protect us and, sometimes, make us vulnerable to severe illness when immune responses get out of control. We also seek to understand what kinds of control measures are most effective in limiting transmission. To understand these issues for even *one* type of disease—say, coronavirus diseases—requires drawing on a wide range of methodologies and disciplines.

We consider evidence from classical *epidemiological* studies of transmission in households and other settings. We consider *immunological* studies that show us how markers of immunity develop, whether they protect us against future disease, and how particular markers (say a certain type of antibody directed at a certain part of the virus) change infection and mortality rates. We consider *molecular genetics* experiments, including those conducted in animal models, that tell us how changes in a virus’s genome affect the course of disease. We consider evolutionary patterns in the virus’s genetic code, seasonal patterns in its transmission and that of other related viruses, and observational studies of the risk factors and circumstances favoring transmission. And, of course, we also consider randomized trials of treatments and prevention measures, when they exist, as we seek to understand which interventions work and which ones may do more harm than good.

The upshot is that, done well, epidemiology synthesizes many branches of science using many methods, approaches, and forms of evidence. No one can be expert in all of these specialties, and few can even be conversant in all of them. But a scientist should be open to learning about all of these kinds of evidence and more.

Thinking about evidence from diverse specialties is critical not only for weighing evidence and deciding how to act but also for developing hypotheses that, when tested, can shed light across specialties. Appropriate humility dictates that molecular virologists should not assume they are experts in social epidemiology, and vice versa. To say “I’m a virologist, so I’m not going to account for any findings from social epidemiology in my work” gives up the chance to understand the world better.

Here’s an example. In the case of a new virus like SARS-CoV-2, the fact that socioeconomically disadvantaged people get sick more often than the wealthy gives clues, which we don’t yet know how to interpret, about the way the virus interacts with hosts. It would be informative to a virologist to distinguish the following two hypotheses (among others): (a) exposure to high doses of virus tends to cause severe disease, and disadvantaged people are often exposed to higher doses due to confined living and working conditions, or (b) comorbidities such as heart disease and obesity are higher among disadvantaged people, and lead to more severe outcomes. Of course, either, both, or neither of these hypotheses may turn out to be important explanations, but the canny virologist should wonder and think about how to distinguish them experimentally and test results against data from human populations. Reciprocally, a canny social epidemiologist should look to virological studies for clues about why COVID-19, like so many other illnesses, disproportionately harms the least advantaged in our society.

In practice, virologists, immunologists, and epidemiologists are different specialists who often work far apart and almost never attend each other’s seminars. I do not think we should spend all our time learning each other’s disciplines. But I do think that a scientist who genuinely wants to solve an important problem should be open to evidence from many sources, should welcome the opportunity to expand their list of hypotheses, and should seek to increase their chances both of making a novel contribution to their field and of being right. Central to this effort is considering information from diverse kinds of studies performed by people with diverse job titles in diverse departments of the university—as well as their diverse forms of data and argumentation.

When we move from the realm of understanding to the realm of intervention, the need for openness to different sources of evidence grows further. In some cases, like whether to use a drug to treat infection or whether to use a mask to prevent transmission, we can draw on evidence from experiments, sometimes even randomized, controlled, double-blind experiments. But in deciding whether to impose social distancing during an outbreak of a novel pathogen—and in thinking about how the course of the epidemic might play out—it would be crazy not to consider whatever data we can, including from mathematical models and from other epidemics throughout history. With infectious diseases, especially new and fast-spreading pandemics, action can’t wait for the degree of evidentiary purity we get from fully randomized and controlled experiments, or from the ideal observational study. At the same time, we must continue to improve our understanding while we act and change our actions as our knowledge changes—leaving both our beliefs and our actions open, as Medawar says, to the reach of criticism and the possibility of modification.

Where does the skepticism so characteristic of the evidence-based tradition come from? One reason may be the habits and heuristics we absorb from textbooks, colleagues, and mentors.

In supervising students and postdocs, inculcating these habits is one of the most challenging, gratifying, and time-consuming parts of scientific training—far more than teaching technical skills. Some of these rules of thumb are well suited to science in general and serve us well throughout our careers, no matter the field. Among these are workaday but important heuristics like: consider alternative hypotheses; look at raw data whenever possible before looking at processed data; and repeat experiments, especially those whose results surprise you. Indeed, these heuristics can be summarized as a form of intense skepticism directed at one’s *own* work and that of one’s team: find all the flaws you can before someone else does; fix those you can and highlight as limitations those which are unfixable. Recently an advanced PhD student said to me: “I read your new idea that you shared on Slack this morning and I’ve been doing my best all afternoon to break it.” It made my day, and made me think I probably had very little left to teach her.

Other heuristics, however, are more specific to a narrow field and may be ill suited to other contexts. Insisting on gold standard, randomized trial evidence *before* prescribing drugs to prevent heart attacks or before performing a certain surgical operation may be a good rule of thumb in medicine (though not all physicians or even philosophers agree). But randomized controlled trials are not available for huge swaths of scientific inquiry, and the narrow populations often studied in such trials can limit their applicability to real-world decision making. Nor are they always available when we need them: they require a lot of time and administrative resources to execute (and money, for that matter). Stumping for Evidence is thus useful in many parts of clinical medicine but impractical in many other aspects of science-informed decision making. Applying this doctrine indiscriminately across all areas of science turns the tools of a specialist into the weapons of a sectarian.

This point was appreciated by David Sackett, one of the pioneers of evidence-based medicine. “Evidence-based medicine is not restricted to randomized trials and meta-analyses,” he wrote in 1996. “It involves tracking down the best external evidence with which to answer our clinical questions” [5]. And in May 2020, on Twitter, the Oxford physician Trisha Greenhalgh, author of one of the most popular textbooks on evidence-based medicine, suggested that in the realm of social interventions to control the spread of COVID-19, the evidence-based clinical paradigm—“waiting for the definitive [randomized controlled trial] before taking action”—“should not be seen as inviolable, or as always defining good science” (https://twitter.com/trishgreenhalgh/status/1256487624346341376)

Indeed, on the question of how we ought to act during an outbreak, two leading epidemiologists in the clinical tradition, Hans-Olov Adami and the late Dimitrios Trichopoulos, argued that the non-interventionist rule of thumb is suitable for chronic, noncommunicable diseases but foolish for fast-moving infectious diseases. In an editorial accompanying an article that showed that the impact of cell phones in causing brain cancer was not large but might be larger than zero, they counseled “cautious inaction” in regulating cell phones. But they noted this is not how you would reason in the case of a transmissible disease:

There is another lesson to be learned about the alarms that have been sounded about public health during the past few years. When the real or presumed risk involves communicable agents, such as the prions that cause bovine spongiform encephalopathy (mad cow disease), no precaution, however extreme, can be considered excessive. By contrast, for noncommunicable agents, such as radio-frequency energy, the lack of a theoretical foundation and the absence of empirical evidence of a substantial increase in risk legitimize cautious inaction, unless and until a small excess risk is firmly documented [6].

I’d tone down the statement that “no precaution, however extreme, can be considered excessive,” which is either rhetorical or sectarian. But in my ideal public health world we’d have a lot more good sense like that proposed by Adami and Trichopoulos, acting not only on the strength of the evidence we have but on the relative harms of being wrong in each direction. And whether waiting or acting, we’d work hard to get the evidence to meet the challenges of skeptics and improve our decision-making, all with an eye to the possibility of criticism and modification Medawar describes.

What does all this mean for the COVID-19 crisis? Scientists of all stripes should work together to improve public health, and none should mistake a professional tendency or a specialist’s rule of thumb for an unshakable epistemological principle. All should support rigorous evidence gathering, especially for the costliest and most disruptive interventions. And insofar as scientists identify with a philosophical school that predisposes them to write off certain forms of evidence entirely, they should, in short, get over it. Instead we should use every possible source of insight at our disposal to gain knowledge and inform decisions, which are always made under uncertainty—rarely more so than at present.
